# rTMS regulates homotopic functional connectivity in the SCD and MCI patients

**DOI:** 10.3389/fnins.2023.1301926

**Published:** 2023-11-23

**Authors:** Honglin Ge, ShanShan Chen, Zigang Che, Huimin Wu, Xinyi Yang, Meizhao Qiao, Lei Chi, Jia Fan, Yeming Zhong, Caiyun Zou, Xingjian Lin, Jiu Chen

**Affiliations:** ^1^Department of Neurosurgery, The Affiliated Brain Hospital of Nanjing Medical University, Nanjing, Jiangsu, China; ^2^Department of Neurology, The Affiliated Brain Hospital of Nanjing Medical University, Nanjing, Jiangsu, China; ^3^Department of Radiology, Nanjing Tongren Hospital, School of Medicine, Southeast University, Nanjing, China; ^4^Department of Human Biology, University of Cape Town Faculty of Health Sciences, Cape Town, South Africa; ^5^Department of Radiology, Nanjing Drum Tower Hospital, Affiliated Hospital of Medical School, Nanjing University, Nanjing, China

**Keywords:** mild cognitive impairment, subjective cognitive decline, repetitive transcranial magnetic stimulation, VMHC, functional magnetic resonance imaging

## Abstract

**Objective:**

Impaired interhemispheric connectivity and corpus callosum atrophy have been linked to cognitive impairment in Alzheimer’s disease (AD). Existing evidence indicates that repetitive transcranial magnetic stimulation (rTMS) targeting the bilateral precuneus may enhance cognitive function in AD. This study aims to investigate the effects of precuneus rTMS on cognitive function, as well as alterations in interhemispheric functional connectivity (FC) and its structural basis in patients with subjective cognitive decline (SCD) and mild cognitive impairment (MCI).

**Methods:**

A total of 14 patients with SCD and 16 patients with MCI were enrolled in this study and received 10 Hz rTMS intervention on the bilateral precuneus for 2 weeks. Neurocognitive scales, structural and functional magnetic resonance imaging were collected at enrollment and after the rTMS intervention. Interhemispheric FC was assessed using mirror homotopic functional connectivity (VMHC), while the structural equation modeling (SEM) was employed to analyze the relationship between corpus callosum volume, interhemispheric connectivity, and cognitive function after rTMS intervention.

**Results:**

The precuneus rTMS not only enhanced episodic memory in SCD, but also improved multiple cognitive domains in MCI. Post-rTMS intervention, decreased VMHC values in the lingual cortex, middle occipital gyrus, putamen, and fusiform gyrus were observed in SCD, and an increased VMHC value in the postcentral gyrus along with reduced VMHC value in the cerebellum and putamen in MCI. After intervention, more brain regions show decreased FC in SCD and MCI patients, suggesting that precuneus rTMS may protect cerebral cortical plasticity by reducing excessive functional compensation, and thus improve cognitive function. The SEM indicated that the corpus callosum serves as the structural foundation for rTMS regulation of interhemispheric FC to further improve cognitive function.

**Conclusion:**

10 Hz rTMS in the bilateral precuneus could be a promising strategy to improve cognitive function in patients with SCD and MCI. Our study implies that improvements in cognition brought about by precuneus rTMS may result from the remodeling of interhemispheric FC, with the corpus callosum possibly acting as the anatomical basis for functional modulation.

## Introduction

Alzheimer’s disease (AD) poses a significant challenge in the field of global neuroscience. The failure of multiple phase III clinical drugs indicates insufficient efficacy of current AD treatments (Kim et al., 2022). The irreversible course of AD underscores the importance of early detection and intervention to stave off AD development and reduce disability mortality. Subjective cognitive decline (SCD) and mild cognitive impairment (MCI) are early manifestations of AD. The characteristic of SCD is subjective perception of memory decline, but objective neurocognitive scales are normal. MCI is a transitional stage between normal aging and mild dementia characterized by mild cognitive impairment but no significant impact on daily life. Approximately 7 and 2.3% of SCD progress to MCI and AD each year (Lista et al., 2015), and approximately 10–20% of patients with MCI progress to AD each year (Petersen, 2004). Therefore, from the perspective of clinical manifestations, it is believed that the progression of AD is the process of SCD developing into MCI and subsequently transforming into AD. During the MCI and SCD stages, the brain already exhibits structural and functional changes typical of AD (Wang et al., 2020). However, the cerebral cortex also displays robust functional compensation (Chen, 2021), presenting an optimal period for targeted early intervention. Non-drug interventions, in comparison to drug trials, are less invasive, safer, more convenient, and likely more acceptable to patients exhibiting mild clinical symptoms of SCD and MCI.

Repetitive transcranial magnetic stimulation (rTMS), a technique frequently employed in clinical neurology research, delivers magnetic signals through the skull with minimal attenuation. This process can potentially induce improvements in neuronal plasticity and neural network reorganization by modulating cortical excitability, metabolism, and cerebral blood flow (Freitas et al., 2011; Qin et al., 2023). The specific mechanism by which rTMS treats cognitive impairment remains undetermined, but it may involve long-term potentiation (LTP), a process where continuous pulse stimulation via rTMS triggers specific neuronal LTP pathways (Muller et al., 2002). Previous studies have shown that LTP is impaired in AD and MCI, and can not only be used as a potential marker to identify AD, but also to predict the clinical progression of dementia (Martorana et al., 2014; Di Lorenzo, 2020). RTMS has a lasting regulatory effect on the excitatory and inhibitory functions of the cerebral cortex. Depending on the frequency of stimulation, rTMS can be categorized as high-frequency stimulation (≥3–5 Hz) or low-frequency stimulation (≤1 Hz; Vucic and Kiernan, 2017). The former may facilitate local neuronal activity and increase excitability in the stimulated brain area, while the latter may inhibit local neuronal activity and decrease excitability in the stimulated brain area. Studies have reported notable enhancements in cognitive function in AD after high-frequency stimulation rTMS course (Yao et al., 2022), indicating that rTMS can effectively ameliorate cognitive impairment.

During the early stages of AD, neuropathological abnormalities and abnormal cortical activation are primarily concentrated in the posterior part of the cerebral cortex. Specifically, the precuneus, posterior cingulate, posterior cortex, and lateral posterior parietal cortex are the first areas to present amyloid plaques and neurofibrillary tangles (Yokoi et al., 2018). Notably, abnormal activation of functional activity in the precuneus cortex has been observed even at the SCD stage (Billette et al., 2022). The precuneus is recognized as a vital hub in the default mode network (DMN), noted for prominent tau pathologic deposition and neuroinflammation (Martorana et al., 2015; Yokoi et al., 2018), and it also serves a crucial vulnerable region for initial episodes of memory impairment in AD (Nilakantan et al., 2017). A recent study assessing rTMS targeting the precuneus suggests that this region plays a role in controlling various aspects of episodic memory, visuospatial processing, and consciousness. It may achieve its effectiveness by modulating the large-scale neural networks it is a part of, e.g., DMN, and through its robust connections with the hippocampus (Chen et al., 2020). This concept was supported by clinical studies, which showed that high-frequency rTMS therapy targeting the precuneus was able to modulate long-term memory function and enhance connectivity between the precuneus and the temporal cortex in healthy participants (Bonnì et al., 2015). A 2-week study on early AD patients using rTMS to target the precuneus found that the cognitive function significantly improved in the group receiving actual stimulation compared to the sham stimulation group (Koch et al., 2022). These research findings suggest that the precuneus could be a promising intervention target to enhance cognitive function within the AD continuum.

Structural and functional magnetic resonance imaging (MRI) serves as bridges between underlying molecular processes and observable symptom behaviors by identifying structural and functional alterations resulting from neuronal and synaptic damage. Voxel-mirrored Homotopic Connectivity (VMHC) can identify changes in functional connectivity between hemispheres by measuring the high degree of synchronicity of spontaneous activity between each voxel in the one hemisphere and the corresponding voxel in the mirror hemisphere (Zuo et al., 2010). Previous studies indicate that the homotopic connectivity changes were associated with pathological markers and disease progression (Chen, 2022), implying that the interhemispheric functional connectivity might be a potential imaging marker of disease severity within the AD continuum (Wang et al., 2015). Previous studies have investigated the mechanism of neural network plasticity, such as the DMN and salience network (Yuan et al., 2023) and the precuneus–hippocampus circuits (Chen et al., 2022). These studies employed rTMS to target the precuneus as a means of improving cognitive function. However, the modulatory effect on interhemispheric functional connectivity and its association with cognitive improvement require further investigation. The corpus callosum plays a significant role in integrating bilateral sensory information and various advanced cognitive functions (Bogen and Bogen, 1988; Paul et al., 2007). Previous studies have demonstrated that corpus callosum atrophy is correlated with cognitive impairment in MCI and AD patients (Pantel et al., 1999). The structural equation model reveals that cognitive impairment in MCI patients is indirectly mediated by changes in functional connectivity between cerebral hemispheres, facilitated by the integrity of the corpus callosum (Chen, 2022). However, the role of rTMS in enhancing cognitive function by modulating changes in interhemispheric functional connectivity and the structural basis of the corpus callosum in this mechanism remain to be clarified.

In this study, we employed multimodal MRI technology to investigate the impact of 10 Hz rTMS on the bilateral precuneus for 2 weeks to improve cognitive abilities. We delved into the role of changes in hemispheric functional connectivity in the mechanisms underlying cognitive improvement. Additionally, we explored the contribution of alterations in hemispheric functional connectivity to the mechanisms responsible for cognitive enhancement. We further probed whether the size of the corpus callosum underpins the ability of rTMS to regulate changes in hemispheric functional connectivity. The combination of rTMS and brain imaging technology will help to understand the role of precuneus target stimulation in regulating cognitive function and the associated interhemispheric connectivity mechanism in SCD and MCI.

## Materials and methods

### Participants

The data for the applied research were obtained from our in-home database: Nanjing Brain Hospital-Alzheimer’s Disease Spectrum Neuroimaging Project 2 (NBH-ADsnp2; Nanjing, China), which is continuously being updated. Information on NBH-ADsnp2 is provided in Supplementary material 1. Thirty-six participants from the NBH-ADsnp2 database participated in this study, including 18 SCD participants and 18 MCI participants. The clinical trial (No. ChiCTR2000034533) was approved by the Medical Research Ethics Committee of the Nanjing Brain Hospital in Nanjing. All participants completed a two-week TMS program (once daily, 5 days per week), with 14 SCD participants and 16 MCI participants ultimately completing the study. Comprehensive neurocognitive assessment, along with structural and functional MRI data, were obtained at baseline and 2 weeks after the TMS course.

The SCD participants met the criteria set forth by the Subjective Cognitive Decline Initiative (SCD-I; Jessen et al., 2014), which are as follows: (1) frequent feeling of memory decline; (2) Subjective Cognitive Decline Questionnaire (SCD-Q) score greater than 5; (3) cognitive performance matched to education and age; and (4) Clinical Dementia Rating Scale (CDR) = 0.

The inclusion criteria of aMCI (Petersen et al., 1999; Winblad et al., 2004) were as follows: (1) memory impairment was reported by participants or confirmed by family members lasting over 3 months; (2) impaired objective memory performance based on one of the following: a. Impairment on two neuropsychological tests of situational memory functioning (≤ 1.0 SD for age-adjusted norms), b. Impairment on neuropsychological tests of cognitive domains of situational memory functioning, visuospatial functioning, executive functioning, and information processing speed (≤ 1.0 SD for age-adjusted norms); (3) CDR = 0.5, Mini-mental State Examination (MMSE) ≥ 24, Activities of Daily Living assessment-20 (ADL-20) ≤ 23, Hamilton Depression Scale (HAMD) ≤ 7; and (4) ability to maintain daily living without dementia.

### Clinical and neuropsychiatric evaluation

All participants underwent comprehensive and standard clinical assessments, as well as neuropsychological tests, at baseline and following the TMS course. Global cognition was evaluated using MMSE, Montreal Cognitive Assessment (MoCA) and CDR. Episodic memory function was evaluated by the Auditory Verbal Learning Test −20 min-delayed recall (AVLT-20-min DR), the Logical Memory Test -20 min delayed recall (LMT-20-min DR), and the Rey Complex Figure Test 20 min delayed recall (CFT-20-min DR). The executive function data were derived from the Category Verbal Fluency Test (VFT) and the Digit Span Test backward (DST-backward). The information processing speed data were obtained from the Digit Symbol Substitution Test (DSST), part A of the Trail Making Test (TMT-A). The visuospatial function data were extracted from the Rey Complex Figure Test (CFT) and the Clock Drawing Test (CDT). The emotional condition of the participants was evaluated using the HAMD and Hamilton Anxiety Scale (HAMA).

### Structural and functional MRI data acquisition

Structural and functional MRI images were undergone using a Siemens 3.0 T singer scanner (Siemens, Verio, Germany) with an 8-channel radiofrequency coil. Three-dimensional T1 weighted images were obtained in a sagittal orientation employing a 3D magnetization-prepared rapid gradient-echo (MPRAGE) sequence utilizing the following parameters: TR = 1,900 ms, TE = 2.48 ms, inversion time (TI) = 900 ms, number of slices = 176, thickness = 1.0 mm, gap = 0.5 mm, matrix = 256 × 256, FA = 9°, FOV = 256 mm × 256 mm, and voxel size = 1 × 1 × 1 mm^3^. The functional images were obtained using a gradient-recalled echo-planar imaging pulse sequence with 240-time points. Repetition time (TR) = 2,000 ms, echo time (TE) = 30 ms, number of slices = 36, thickness = 4.0 mm, gap = 0 mm, matrix = 64× 64, flip angle (FA) = 90°, field of view (FOV) = 220 mm × 220 mm, acquisition bandwidth = 100 kHz, and voxel size = 3.4 × 3.4 × 4 mm^3^.

### Intervention-rTMS treatment

All participants with SCD and MCI were given rTMS stimulation targeting the bilateral precuneus using a Magstim Rapid2 magnetic stimulator with a 70 mm figure-8-shaped coil. The intersection tip of two coil rings was placed at the Pz site to stimulate the Precuneus with the assistance of a 10–20 electroencephalogram system. The rTMS stimulation frequency was 10 Hz (Chen et al., 2020, 2022; Yuan et al., 2023). The Resting State Motor Threshold (RMT) is a measure obtained by stimulating the left motor cortex with the right hand’s First Dorsal Interosseous (FDI) muscle in a relaxed state, causing at least 5 of the 10 single pulse stimuli recorded on electromyography to have a motor evoked potential amplitude greater than 50 μV, or at least 5 out of 10 single pulse stimuli to result in FDI twitching in the right hand. The stimulation intensity of rTMS is set to 100% of the RMT. A series of stimuli, consisting of 40 instances each lasting 4 s and spaced 56 s apart, is repeated 25 times for a total of 1,000 pulses. Participants receive rTMS treatment while sitting comfortably in a lounge chair and receiving guidance from a therapist. Suitable headrests are provided to stabilize the participants’ heads. The treatment lasts for approximately 25 min, once a day, 5 days a week, over 2 weeks. All participants underwent comprehensive neuropsychological evaluations and structural as well as functional magnetic resonance imaging scans before treatment and 2 weeks after treatment. None of the participants reported any adverse effects or discomfort during the TMS treatment period.

### Image preprocessing

The structural and functional images are preprocessed by Processing Assistant for resting-state fMRI (DPARSF)^1^ and Statistical Parametric Mapping (SPM12).^2^

### Preprocessing of structural MRI

Structural images were preprocessed using the Data Processing and Analysis of Brain Imaging (DPABI) based on the Statistical Parametric Mapping 12 (SPM 12) (see footnote 2) program, which was implemented in MATLAB2013b.^3^ Original DICOM format images were converted to the NIFTI format and spatially normalized. Then, the gray matter, white matter, and cerebrospinal fluid images, obtained by segmenting the MPRAGE images, were standardized to the Montreal Neurological Institute (Montreal Neurological Institute, MNI) standard space (1.5 mm × 1.5 mm × 1.5 mm). Subsequently, the smoothing kernel with a full width at half maximum (FWHM) of 6 mm × 6 mm × 6 mm was used to obtain maps of the gray matter and white matter. For subsequent analyses, the obtained voxel-wise gray matter volume maps were resampled to a setting of 3 × 3 × 3 mm3 voxels, and voxel-based gray matter volume correction.

To obtain the volume of corpus callosum, a prior region of interest (ROI) of the corpus callosum was created as a structural mask using the WFU PickAtlas Tool (The Functional MRI Laboratory, Wake Forest University School of Medicine, Winston-Salem, NC, United States).^4^

### Preprocessing of resting-state fMRI data

Preprocessing of resting-state fMRI data Resting-state fMRI data were preprocessed using DPABI implemented in MATLAB2013b (see footnote 3). We discarded the first 10 volumes and performed slice timing correction and head movement correction. Images of a subject were excluded if the translation or rotation exceeded 3 mm and 3^°^. Then, images were spatially normalized to the MNI echo-planar imaging template and resampled to a default setting (3 × 3 × 3 mm^3^ voxels). And nuisance covariate regression of 24 motion parameters, global signal, white matter signal, and cerebrospinal fluid signal were performed. Finally, a 6 × 6 × 6 mm FWHM were used to reduce high spatial frequency noise and the filtering frequency was 0.01–0.1 Hz (Chen, 2022).

All normalized T1 images were averaged to generate a mean normalized T1 image to obtain VMHC maps. Then, a group-specific symmetrical template was created by averaging left–right symmetric version of this mean image. Finally, homotopic FC was computed between any pair of symmetric inter-hemispheric voxels. Subsequently, the Pearson’s correlation coefficient was computed between the residual time series of each voxel and its contralateral hemispheric counterpart. Finally, these correlation values were performed by the Fisher Z-transformed.

### Statistical analysis

Statistical analysis was performed by using the Statistical Package for the Social Sciences (SPSS) software version 25.0 (IBM, Armonk, New York, NY, United States). Paired t-test analysis was conducted to compare the neuropsychological scales before and after the 2-week rTMS treatment to evaluate the improvement effect of rTMS on cognitive function in SCD and MCI participants. Value of *p* < 0.05 was considered statistically significant.

Paired t-tests were conducted pre- and post- rTMS intervention to obtain voxel-based comparisons of VMHC maps, in the SCD and MCI groups, respectively. Among the above statistics, *p* < 0.001 and voxel ≥60 are considered to have statistical differences. Pearson correlation analysis is used for the correlation analysis of the average time series of the region of interest. Pearson correlation analysis was performed to examine the relationship between VMHC index, corpus callosum volume, and neuropsychological scales in SCD and MCI participants. These analyses controlled for age, sex, education, and total intracranial volume as covariates. *p* value <0.05 is considered statistically significant.

We conducted the structural equation modeling (SEM) using AMOS 24.0.0 software (Meadville, PA, United States; Crowley and Fan, 1997). SEM is a statistical method for analyzing causal relationships between variables. The construction of SEM in this study is based on the hypothesis that the corpus callosum serves as the structural basis for rTMS to regulate interhemispheric FC and improve cognitive function. We use SEM to explore the relationship between corpus callosum volume, homotopic FC, and cognitive function. Specifically, corpus callosum volume, VMHC index, and episodic memory scores are used as observation variables (represented by rectangles), with corpus callosum volume and VMHC index as endogenous variables and episodic memory scores as exogenous variables. Exploratory factor analysis is used to determine whether all free parameters have unique solutions, and the maximum likelihood estimation algorithm is used to estimate the parameters in the model. Finally, we utilized the following indices to evaluate the goodness of fit: Chi-square/degrees of freedom <3.0, goodness of fit index (GFI) < 0.9, adjusted goodness of fit index (AGFI) < 0.9, and Root Mean Square Error of Approximation (RMSEA) < 0.05. Two tailed significance *p* value <0.05 was considered significant.

## Results

### Characteristics of demography and neuropsychological scales

**Figure 1:**
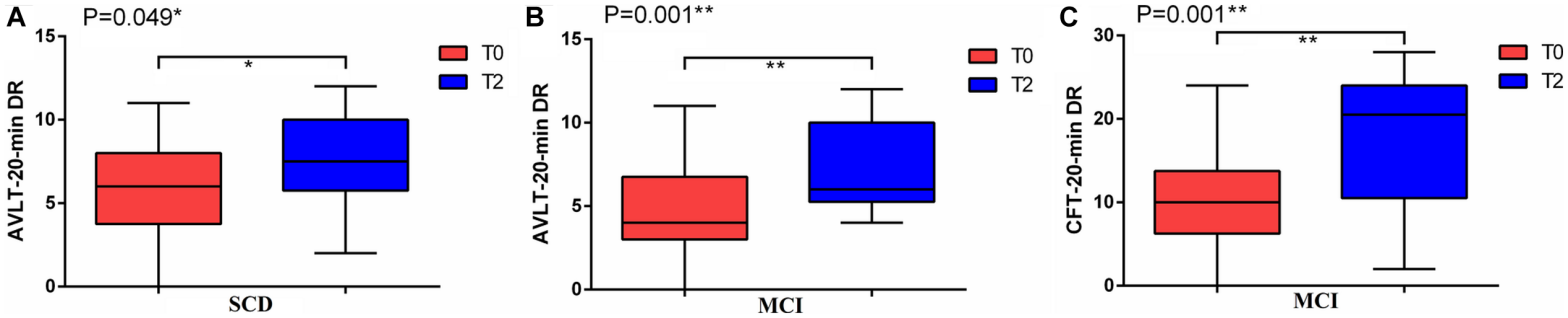
Mean cognitive performance at baseline (T0) and after 2 weeks of treatment (T2) in the rTMS groups. **(A)** AVLT-20-min DR in SCD; **(B)** AVLT-20-min DR in MCI; **(C)** CFT-20-min DR in MCI.

A total of 30 participants completed the experiment, including 14 SCD and 16 MCI participants. Table 1 summarized the demographic and clinical characteristics of 30 participants in two groups. In the SCD group, paired t-test results indicated a significant increase in the AVLT-20-min DR after 2 weeks of precuneus rTMS (PCUN-rTMS) treatment (T2) compared to baseline (T0; *p* < 0.05; Figure 1). However, the scores for MOCA, LMT-20-min DR, SCD-Q, CDR and CFT-20-min DR were not significantly different between T0 and T2. In the MCI group, paired t-test results showed that the scores for AVLT-20-min DR, CFT-20-min DR, DSST, CFT, HAMD and HAMA scores were significantly higher at T2 than at T0 following the PCUN-rTMS course (*p* < 0.05; Figure 1). There was no significant difference in the MOCA, LMT-20-min DR, SCD-Q, and CDR scores between T0 and T2 after the Precuneus rTMS treatment.

**Table 1:** Demographics and neuropsychological assessment of SCD and MCI subjects.

	SCD (*n* = 14)			MCI (*n* = 16)		
	T0	T2	*p*	T0	T2	*p*
Age	67.21 ± 6.90	67.21 ± 6.90	–	65.94 ± 7.03	65.94 ± 7.03	–
Female	11	11	–	14	14	–
Education	11.75 ± 2.94	11.75 ± 2.94	–	12.5 ± 2.76	12.5 ± 2.76	–
MMSE	28.29 ± 1.44	28.43 ± 1.29	0.655	27.69 ± 1.40	28.13 ± 1.17	0.387
MOCA	25.57 ± 1.84	25.79 ± 2.40	0.732	23.88 ± 2.23	24.81 ± 3.68	0.243
CDR	0.43 ± 0.18	0.36 ± 0.23	0.1665	0.38 ± 0.22	0.28 ± 0.26	0.333
AVLT-20-min DR	5.71 ± 2.68	7.29 ± 2.60	0.049^*^	4.56 ± 3.00	7.25 ± 2.63	0.001^*^
LMT-20-min DR	3.86 ± 2.61	5.79 ± 3.95	0.182	4.00 ± 2.87	5.25 ± 2.63	0.159
CFT-20-min DR	18.79 ± 4.23	20.07 ± 6.43	0.450	10.44 ± 6.37	18.19 ± 7.38	0.001^**^
VFT	20.5 ± 3.64	21.64 ± 4.42	0.411	25.19 ± 28.08	18.19 ± 2.94	0.373
DST-backward	12.79 ± 1.37	13.43 ± 3.20	0.456	11.25 ± 1.30	12.13 ± 1.65	0.074
DSST	39.8 ± 10.19	43.21 ± 14.19	0.186	32.44 ± 6.37	38.06 ± 9.74	0.032^*^
TMT-A	52.64 ± 10.74	56.43 ± 16.71	0.321	64.06 ± 18.71	58.06 ± 13.80	0.074
CFT	35.36 ± 1.59	35.36 ± 1.11	1.000	32.56 ± 3.94	34.25 ± 1.92	0.041^*^
CDT	9.29 ± 0.96	9.07 ± 0.70	0.385	9.13 ± 0.93	9.25 ± 0.66	0.652
HAMD	4.36 ± 4.05	3.14 ± 3.81	0.405	3.44 ± 2.34	12.00 ± 1.73	0.012^*^
HAMA	5.43 ± 6.04	4.71 ± 4.60	0.609	4.00 ± 2.03	1.75 ± 1.35	0.002^*^
SCD-Q	6.29 ± 0.84	6.07 ± 1.10	0.481	5.72 ± 1.29	5.38 ± 1.52	0.127

Numbers are given as means (standard deviation, SD) unless stated otherwise. Paired t-tests were used to test for statistical differences between groups before and after the TMS intervention. *showed a significantly group difference between T0 and T2, *p* < 0.05; SCD, subjective cognitive decline; MCI, mild cognitive impairment.

### Comparisons of VMHC values between pre- and post-rTMS

**Figure 2:**
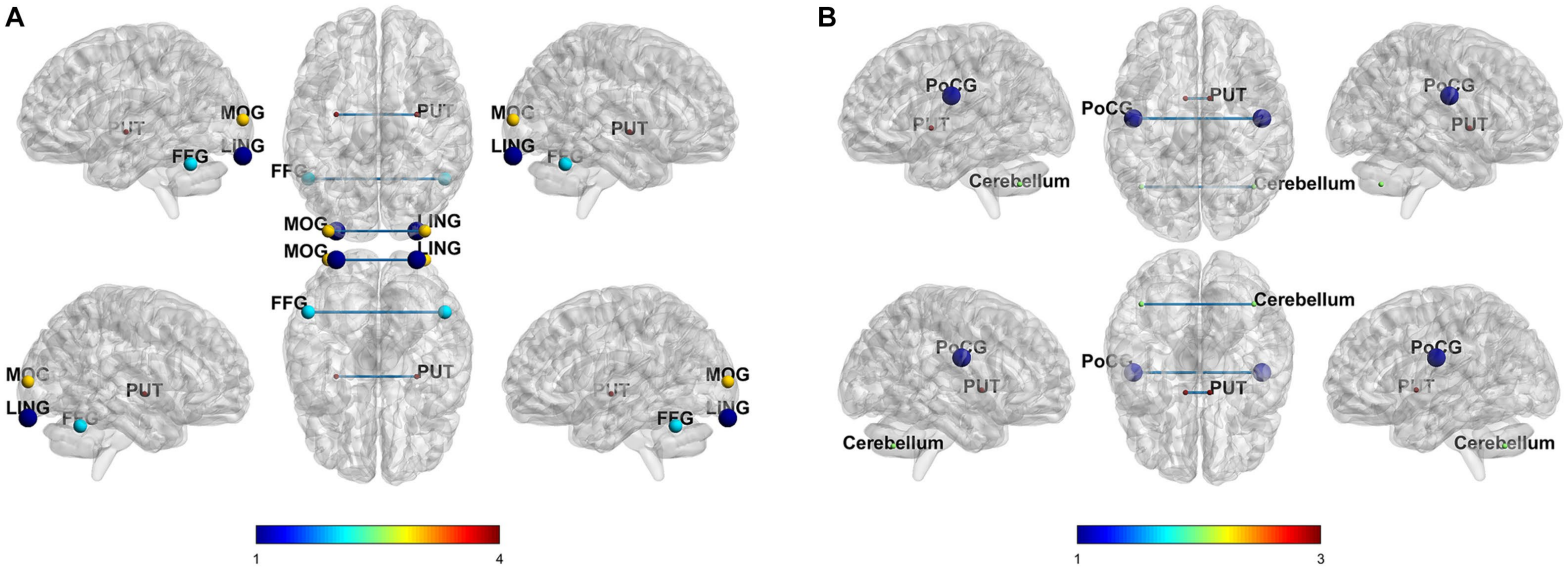
Comparisons of VMHC values between pre- and post-rTMS of SCD **(A)** and MCI **(B)**.

In the SCD group, compared to the T0, T2 condition revealed decreased VMHC value in the lingual cortex, middle occipital gyrus, putamen, and fusiform gyrus. In the MCI group, compared to the baseline state, 2 weeks of treatment condition revealed enhanced VMHC value in the postcentral gyrus, and decreased VMHC value in the cerebellum and putamen. The correction methods for the paired t-test mentioned above are AlphaSim, voxel *p* < 0.001, cluster size >60 voxels, and cluster *p* < 0.05. The results are shown in Figure 2 and Table 2.

**Table 2:** VMHC differences in brain regions in SCD and MCI patients.

Region (aal)	Peak MNI coordinate x y z			T cluster number	
SCD T2<T0 rTMS					
B Lingual gyrus	±30	−96	−21	−5.06	157
B Fusiform gyrus	±51	−57	−27	−4.25	132
B middle occipital gyrus	±36	−96	6	−6.05	127
B Putamen	±30	−9	−3	−6.62	96
MCI T2 > T0 rTMS					
B Postcentral gyrus	±48	−12	24	3.68	91
MCI T2<T0 rTMS					
B Cerebellum	±42	−63	−42	−3.54	64
B Putamen	±9	3	0	−3.84	64

The x, y, z coordinates are the primary peak locations in the MNI space. Cluster size > 60 voxels in paired t test, AlphaSim corrected, voxel *p* < 0.001, and cluster *p* < 0.05 corected; B bilaterally; SCD, subjective cognitive decline; MCI, mild cognitive impairment.

### Behavioral significance of changes in VMHC values between pre- and post-rTMS

**Figure 3:**
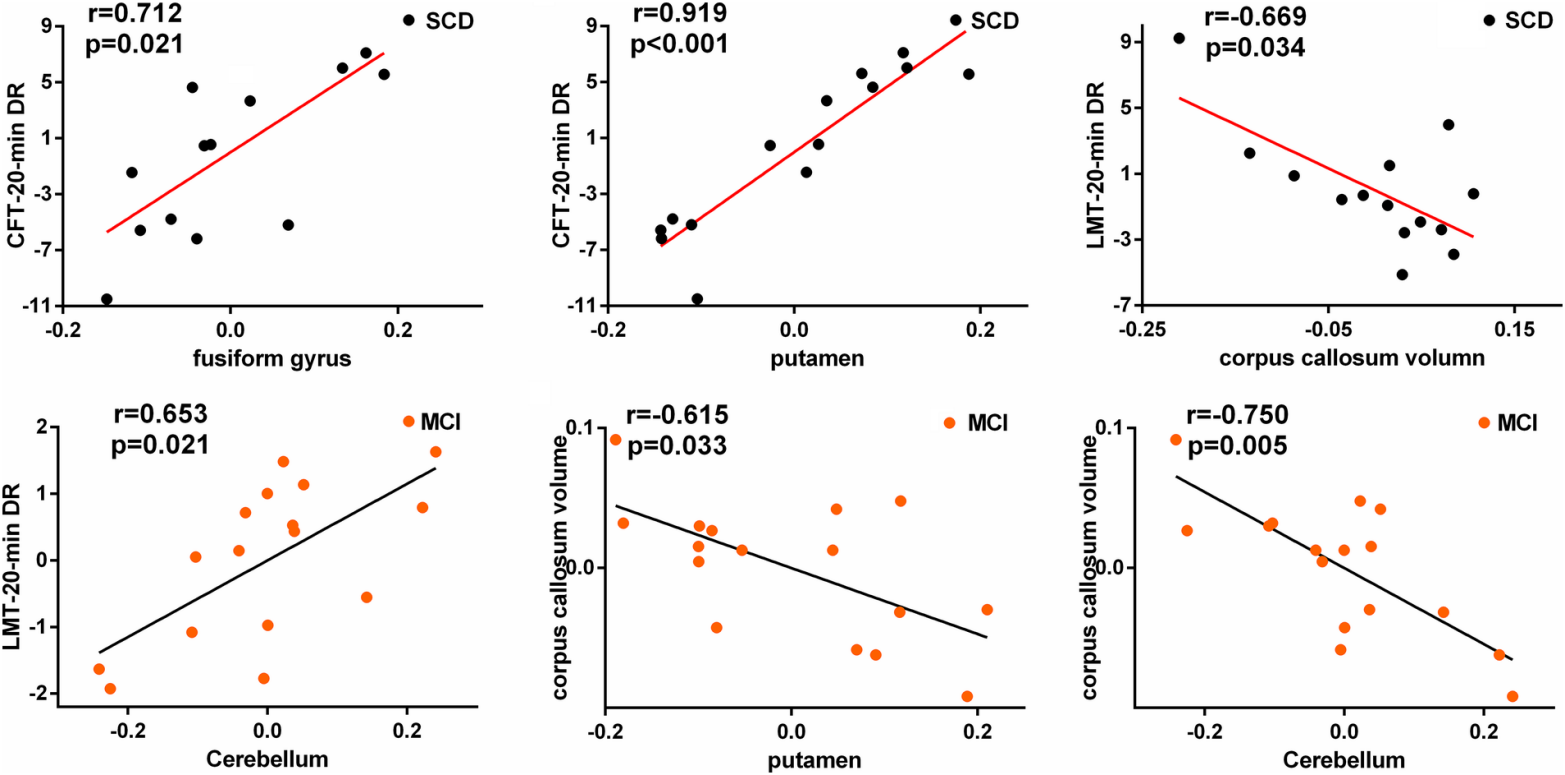
Significant associations between altered homotopic connectivity and cognitive function as well as corpus callosum volume after rTMS intervention in SCD and MCI patients.

Pearson correlation analysis was performed between decreased and increasing VMHC values and neuropsychological scales, and corpus callosum volume (Figure 3). In the SCD group, the CFT-20-min DR scores were showed a positive correlation with altered VMHC values in the fusiform gyrus (*r* = 0.712, *p* = 0.021), the lingual gyrus (*r* = 0.734, *p* = 0.016), and putamen (*r* = 0.919, *p* = 0.000). Moreover, there was a negative correlation between the corpus callosum volume and altered VMHC values in the cerebellum (*r* = −0.628, *p* = 0.052) and LMT-20-min DR (*r* = −0.669, *p* = 0.034). In the MCI group, altered VMHC values in the cerebellum showed a positive correlation with LMT-20-min DR (*r* = 0.653, *p* = 0.021), while the corpus callosum volume was negatively correlated with altered VMHC values in the putamen (*r* = −0.615, *p* = 0.033) and cerebellum (*r* = −0.750, *p* = 0.005). All correlation analyses were controlled for age, sex, education level, and total intracranial volume. FDR correction was used for multiple comparisons (*p* < 0.05).

### The hypothesized model of the structural basis of rTMS stimulation

**Figure 4:**
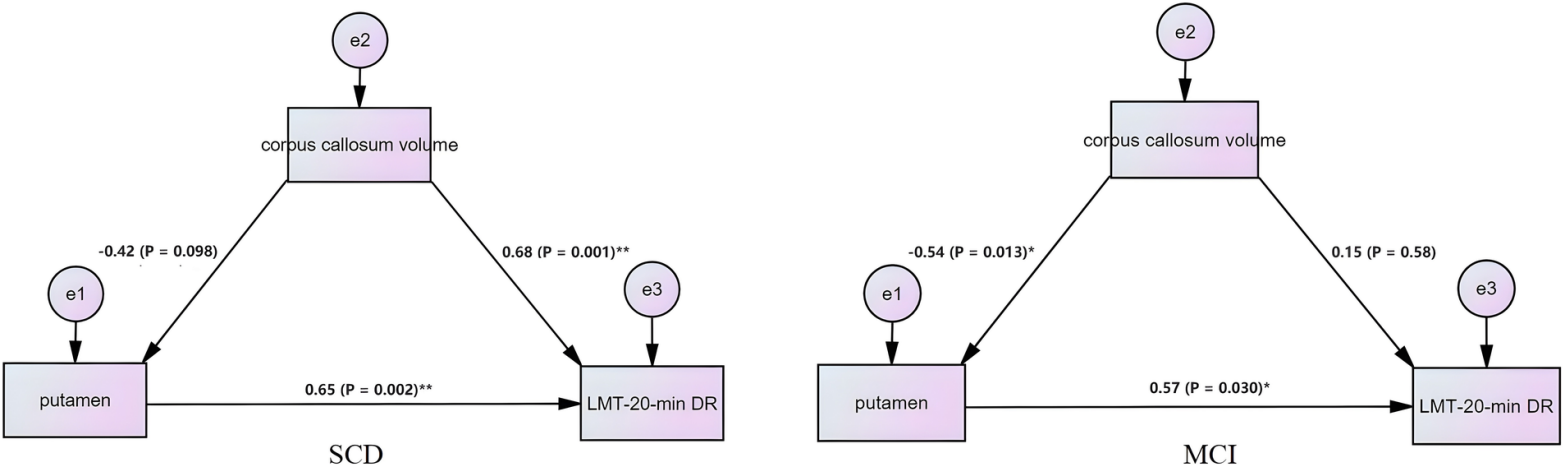
Structural equation model reveals the causal relationship among interhemispheric functional connectivity, episodic memory, and corpus callosum volume. The square represents the observed variable and the ellipse represents the latent variable.

This study used SEM to investigate changes in corpus callosum volume and interhemispheric FC and their effects on cognition in SCD and MCI patients. Our results show that the corpus callosum is the structural basis for the modulation of interhemispheric FC by PCUN-rTMS, resulting in cognitive improvement in patients with SCD and MCI. The SEM model construction of the putamen FC, corpus callosum, and episodic memory function showed statistical significance (Figure 4), while other brain regions showed no significance. Mediation analysis in the SCD group indicated that changes in putamen FC after 2 weeks of rTMS treatment, mediated in part by corpus callosum volume, resulting in changes in episodic memory. In the MCI group, mediation analysis revealed FC changes in the putamen, partially mediated indirectly by corpus callosum volume, resulting in episodic memory changes after 2 weeks of rTMS treatment. In the above analysis, age, gender, education level and total intracranial volume were removed as covariates.

## Discussion

In our study, we examined the impact of 2-week rTMS that targets the precuneus on cognitive function in SCD and MCI participants. We investigated the properties of hemispheric connectivity associated with an enhancement in episodic memory and aimed to unravel the complex effects of rTMS on cognitive function enhancement. Our preliminary findings suggest that rTMS, when targeted at the precuneus, can notably enhance the episodic memory domains in SCD patients, and multi-domain cognitive performance in MCI patients, including episodic memory, information processing speed, visuospatial function, and emotional condition. Additionally, cognitive function can be improved through rTMS by modulating the plasticity of inter-hemispherical FC in both SCD and MCI participants. Finally, our SEM analysis suggests that the volume of the corpus callosum serves as the structural basis allowing rTMS to regulate changes in interhemispheric FC, leading to improvements in cognitive impairment. Our findings, which utilize rTMS targeting the precuneus alongside multimodal MRI data, provides insights into the effect of rTMS on cognitive enhancement in MCI and SCD patients. This may support the potential of precuneus targeting as a promising approach to improving cognition in individuals with neurodegenerative diseases.

Previous research has demonstrated that rTMS protocols aiming at either the left DLPFC or bilateral cerebellum positively affect general cognition in early AD patients (Simko et al., 2022; Yan et al., 2023). There is currently no agreement on the effectiveness of rTMS targeting the precuneus in individuals with preclinical AD. Given that the precuneus, as the hub of the DMN, exhibits cortical dysfunction in early stages of degenerative disease (Nilakantan et al., 2017), magnetic stimulation of this region could provide an innovative multi-target intervention strategy for cognitive preservation in SCD and MCI patients. Our findings indicate that a PCUN-rTMS intervention positively modulated cognitive function in MCI and SCD patients after 2 weeks of treatment. Specifically, improvements were observed primarily in episodic memory in SCD patients, whereas MCI patients demonstrated enhancements in overall cognitive function, including memory, information processing speed, visuospatial function, and emotional processing. Our findings support previous research, which illustrated that rTMS interventions targeted at the precuneus can enhance cognitive functions, notably episodic memory, in SCD and MCI patients (Chen et al., 2020, 2022). Similar findings were obtained in healthy controls, indicating that the PCUN-rTMS course can modulate long-term memory function and enhance connectivity between the precuneus and temporal cortex (Mancini et al., 2017). A randomized double-blind controlled trial, published in the journal BRAIN, demonstrated a significant cognitive improvement in the actual stimulation group after a 2-week PCUN-rTMS treatment regimen in early AD patients. Building upon this, the study introduced a 22-week maintenance treatment, revealing that PCUN-rTMS could significantly delay long-term cognitive decline in AD patients (Koch et al., 2022). Our findings are further supported by another randomized double-blind study, which demonstrates that precuneus stimulation can selectively enhance episodic memory in patients with early AD (Koch et al., 2018). Additionally, the mean age of SCD participants in the study was older than that of MCI participants, which may be attributed to the fact that the subjects were recruited from the community and had mild cognitive impairment, while elderly MCI patients were more inclined to seek hospital assistance due to more severe cognitive impairment. Young AD patients exhibit different patterns in structure and metabolic trajectory (Chiaravalloti et al., 2016). Furthermore, the limited sample size of the study may also account for this phenomenon. In conclusion, our preliminary results suggest a new intervention strategy for rTMS intervention in degenerative diseases: the precuneus appears to be a potentially effective intervention target for individuals with SCD and MCI.

Cognitive changes induced by rTMS may be due not only to modulation of functional brain activity in the stimulated region, but also to indirect effects on other relevant or distant brain regions (Rossi et al., 2001). Prior studies have demonstrated that when the precuneus is stimulated via rTMS, cognitive function can be enhanced through the regulation of brain circuits’ plasticity and the reorganization of brain networks; for example, the regulation of precuneus-hippocampal circuits and the salience network (Chen et al., 2020; Yuan et al., 2023). The brain’s lateralization in neurodegenerative diseases is linked to cognition and disease progression, indicating that changed connectivity between the hemispheres could be a marker of cognitive decline (Stefanits et al., 2012; Frings et al., 2015). Unfortunately, the mechanisms behind interhemispheric FC alterations and cognitive enhancement following rTMS treatment remain underexplored in preclinical AD patients. Thus, in this study, we investigated the effects of precuneus-focused rTMS on interhemispheric FC in individuals with SCD and MCI, and analyzed the correlation between changes in this FC and cognitive improvement.

Our findings reveal that following 2 weeks of rTMS intervention, SCD demonstrated a decrease in interhemispheric functional connectivity in the lingual gyrus, fusiform gyrus, putamen, and the middle occipital gyrus. The lingual gyrus and middle occipital gyrus reside in the occipital lobe, which facilitates not only visuospatial processing but also object and face recognition as well as memory formation (Rehman and Al Khalili, 2020). A study of brain connectivity investigating the structure of white matter in the precuneus has identified five intra- and inter-hemispheric anatomical connections. These connections potentially have a function in the integration of information from higher-order brain networks, including the DMN (Tanglay et al., 2022). The initial microanatomy being examined concerns the precuneus’s short connection to the superior parietal lobule and occipital cortex, which may function primarily in visual memory. This anatomical foundation can give rise to a plausible hypothesis explaining our results: magnetic stimulation of the precuneus travels through the white matter anatomical pathway connecting the precuneus and occipital cortex, leading to an impact on interhemispheric FC within the occipital cortex, notably the lingual and middle occipital gyrus. Further correlation analysis demonstrated a significant correlation between the interhemispheric functional connectivity changes of the lingual gyrus and CFT-20-min DR scores, which supported our hypothesis. The fusiform gyrus, positioned between the inferior temporal gyrus and the parahippocampal gyrus, is crucial in processing color information, facial and bodily recognition, word recognition, and categorization (Weiner et al., 2018). The fusiform and precuneus are part of the DMN (Liao et al., 2018), and stimulating the precuneus induces FC changes within the DMN, which could lead to interhemispheric FC changes in the fusiform. Previous research has shown that individuals with SCD have higher concordance and FC in the fusiform gyrus compared to healthy older adults. This increased connectivity is considered a compensatory mechanism in SCD individuals (Chen et al., 2020; Yang, 2020). The putamen, a subcortical structure, mainly located in the basal ganglia, is involved in learning and motor control, including speech articulation and language function, reward, cognitive function, and addiction (Ghandili and Munakomi, 2020). Notably, a decrease in interhemispheric FC is closely associated with cognitive improvement, suggesting that subcortical structures play a significant role in cognitive function. Interestingly, rTMS modulation in SCD participants resulted in a decrease, rather than an increase, in interhemispheric connectivity. The possible mechanism is that the increased functional connectivity in SCD patients is a compensatory effect, representing the excessive activation of neuronal cells and the deposition of more pathological markers (Mashour et al., 2019). RTMS stimulation regulates this abnormal compensation and restores it to normal functional connectivity, thereby regulating cognitive function.

Our study found coexisting increased and decreased FC after rTMS intervention for MCI patients, with decreased FC in the cerebellum and putamen areas of the brain and increased FC in the postcentral gyrus. The postcentral gyrus (DiGuiseppi and Tadi, 2023) is located on the side of the parietal lobe and contains the primary somatosensory cortex, which is an important brain region responsible for proprioception. This area perceives various peripheral sensations of the body, including touch, pressure, temperature, and pain. A recent study has found that MCI patients exhibit overestimation of the angle turn in the outbound path and more variable inbound distances and directions compared with healthy elderly (Castegnaro et al., 2023), indicating that MCI patients exhibit abnormalities in information processing in the peripheral sensory-central somatosensory cortex. Our study found that MCI patients showed an increased VMHC of the postcentral gyrus after rTMS intervention, which may be a neural loop mechanism for improving visual spatial function in MCI patients. A contralateral intermittent theta burst stimulation (iTBS) intervention in healthy individuals found an increase in dynamic functional connectivity of postcentral gyrus in post TBS, supporting our research conclusion (Zhang et al., 2019). More and more evidence suggests that the cerebellum plays an important role in a wide range of functions such as cognition, emotional processing, and social behavior (Schmahmann and Caplan, 2006; Ferrari et al., 2023). The rTMS study targeting the cerebellum has achieved effective improvements in cognitive function in early AD patients (Yao et al., 2022; Yan et al., 2023). We found that after PCUN-rTMS, the cerebellar FC presented decreased in MCI patients. Although we do not have a suitable explanation for this phenomenon, similar to the decreased FC in putamen, it may be due to the fact that our subjects were recruited from the community and they were in the very early stages of MCI. Therefore, similar to the results of SCD, TMS improves cognitive function by regulating overactivated brain regions, and the correlation between the decreased cerebellar and putamen FC and episodic memory scores may support this hypothesis.

The corpus callosum is a critical anatomical structure of interhemispheric connectivity that supports a wide range of cognitive, behavioral and neural functions in the human brain (Bogen and Bogen, 1988). Previous research has indicated that corpus callosum shrinkage is linked with cognitive decline in MCI and AD patients (Pantel et al., 1999; Pogarell et al., 2005). This suggests that corpus callosum volume can serve as an imaging marker for neurodegenerative diseases. An fMRI study conducted on patients with progressive MCI revealed that the volume of the corpus callosum affected not only interhemispheric FC changes directly, but also indirectly caused cognitive changes via alterations in interhemispheric FC (Chen, 2022). Based on these findings, we investigated the influence of corpus callosum volume on interhemispheric effects post-TMS intervention in individuals with SCD and MCI. Our study discovered that corpus callosum volume was linked to interhemispheric FC after rTMS intervention, indicating that the corpus callosum could be a structural foundation for the impact of rTMS stimulation on interhemispheric connectivity. Further SEM findings confirmed our hypothesis that the changes in interhemispheric FC after rTMS intervention were partially influenced by the corpus callosum structure. This study provides preliminary results, offering novel insights into the anatomical basis of FC variations pre- and post-rTMS intervention. These findings provide supporting evidence for further exploration into the mechanisms of TMS.

## Conclusion

The results of the study suggest that applying 10 Hz rTMS on bilateral precuneus may be a promising method for treating patients with prodromal AD. This treatment not only demonstrated improvements in episodic memory function but also in visuospatial, and emotional functions. Through investigating previously established neural circuits, our findings offer evidence that the modulation of interhemispheric FC may play a crucial role in the neuroimaging mechanism of PCUN-rTMS for enhancing cognition. Additionally, the research suggests that the volume of the corpus callosum may serve as the anatomical foundation for PCUN-rTMS modulation of functional connectivity between cerebral hemispheres.

### Limitations

Although the results currently appear promising, it is important to acknowledge certain limitations that need to be considered. One major limitation of our study is the lack of a sham stimulation group. Initially, we planned to include a sham rTMS group targeting the precuneus, but due to limited participant numbers and resulting insufficient statistical power, this was not feasible. It is imperative that this issue be addressed in future studies. Secondly, we did not investigate the long-term effects of rTMS on the maintenance of cognitive improvement and interhemispheric connectivity changes. It would be valuable for future research to examine the sustained effects of rTMS over an extended period of time. In addition, our subjects were recruited from the community, thus lacking invasive pathophysiological biomarkers for AD pathology. Finally, one drawback is the lack of neuronavigation in rTMS targeting the precuneus. In fact, the subjects enrolled in our research group since August 2023 have all used neuronavigation for positioning. Addressing these limitations in future studies will help to further advance our understanding of the effects and potential applications of rTMS in cognitive interventions.

## Data availability statement

The raw data supporting the conclusions of this article will be made available by the authors, without undue reservation.

## Ethics statement

The studies involving humans were approved by Human Participants Ethics Committee of the Affiliated Brain Hospital of Nanjing Medical University. The studies were conducted in accordance with the local legislation and institutional requirements. The participants provided their written informed consent to participate in this study.

## Author contributions

HG: Formal analysis, Software, Writing – original draft. SC: Conceptualization, Writing – original draft. ZC: Methodology, Writing – original draft. HW: Project administration, Writing – original draft. XY: Validation, Writing – original draft. MQ: Visualization, Writing – original draft. LC: Supervision, Writing – original draft. JF: Data curation, Writing – original draft. YZ: Investigation, Writing – original draft. CZ: Supervision, Writing – original draft. XL: Funding acquisition, Resources, Writing – review & editing. JC: Funding acquisition, Resources, Writing – review & editing.

## Footnotes

^1^ http://restfmri.net/forum/DPARSF

^2^ http://www.fil.ion.ucl.ac.uk/spm

^3^ http://www.mathworks.com/products/matlab/

^4^ http://www.ansir.wfubmc.edu
