# Supplementary material for: rTMS regulates homotopic functional connectivity in the SCD and MCI patients
Source: Front Neurosci. 2023 Nov 23;17:1301926. doi: 10.3389/fnins.2023.1301926 (PMC10702213; doi:10.3389/fnins.2023.1301926)
Supplement: Supplementary file 1 [file Data_Sheet_1.doc]

# NBH-ADsnp-2 database

Data used in this study were obtained from the Nanjing Brain Hospital-Alzheimer’s Disease Spectrum Neuroimaging Project Version 2 (NBH-ADsnp-2) database. NBH-ADsnp-2 is an upgrade of NBH-ADsnp and is derived from an Alzheimer’s Disease Spectrum Neuroimaging cooperative Project that was jointly built in September 2022 by Department of Radiology, Nanjing Drum Tower Hospital and Department of Radiology and Neurology, the Affiliated Brain Hospital of Nanjing Medical University. Prof. Jiu Chen, PhD, MD, from Nanjing Drum Tower Hospital, and Xingjian Lin, MD and Chaoyong Xiao, MD, from the Affiliated Brain Hospital of Nanjing Medical University, acts as the principal investigator of NBH-ADsnp-2. NBH-ADsnp-2 was initiated by Dr. Jiu Chen, Dr. Xingjian Lin, and Dr. Chaoyong Xiao and was named by Dr. Chen, Dr. Lin, Dr. Xiao's cooperative research group (discussed by Chen Xue, Guan-jie Hu, Wen-wen Xu, Wan Liu, Wen-zhang Qi, Si-yu Wang, Jia-ni Xu, Shan-shan Chen, Honglin Ge, Zheng Yan, Yu Song, Qianqian Yuan, Huimin Wu, Xuhong Liang, Xinyi Yang and finally verified by Jiu Chen, Xingjian Lin, and Chaoyong Xiao). NBH-ADsnp-2 is an observational and intervention study which includes cross-sectional and longitudinal follow-up components. The goal of NBH-ADsnp-2 is to identify early neuroimaging biomarkers of preclinical Alzheimer’s Disease (AD) spectrum {subjective cognitive decline (SCD), amnestic mild cognitive impairment (aMCI), amnestic mild cognitive impairment (naMCI), and AD}, to predict the disease progression of individuals within preclinical AD spectrum, and to provide imaging-based targets for individualized intervention in order to prevent the disease deterioration from preclinical stages to the eventually progressed AD. All subjects in NBH-ADsnp-2, who were all Han Chinese and right-handed, were recruited initially from hospitals and local communities by advertising and by means of broadcasting. This database used a standardized clinical evaluation protocol that included a medical history interview, neurologic examination, a battery of neurocognitive assessment, and resting-state MRI scan (T1, T2, 3D T1, DTI, and BOLD) for all participants (healthy controls, SCD, naMCI, aMCI, and AD). In addition, MRI data collected after 2 and 4 weeks of rTMS intervention in patients with SCD and MCI were added after the database upgrade. All subjects and their study partners completed the informed consent process, and the study protocols were reviewed and approved by the responsible Human Participants Ethics Committee of the Affiliated Brain Hospital of Nanjing Medical University (No. 2018-KY010-01, No. 2020-KY010-02, No.2021-KY029-01, No. 2021-KY009-01, No. 2022-KY042-01, No. ChiCTR2000034533, No. ChiCTR1900022287).

The general eligibility, inclusion, and exclusion criteria for NBH-ADsnp-2 subjects can be found as follows:

Inclusion criteria of SCD subjects were identified meeting the published SCD research criteria proposed by the Subjective Cognitive Decline Initiative (SCD-I) , and the detailed inclusion criteria have been described in our previously published studies , as follows: (a) self-reported persistent memory decline, which was confirmed by an informant; (b) Subjective Cognitive Decline Questionnaire (SCD-Q) score > 5 ; (c) performance within the normal range on MMSE and MoCA (adjusted for age and education); (d) Clinical Dementia Rating (CDR) = 0; and (e) subjects aged between 50 and 80 years old.

Inclusion criteria of naMCI subjects were referenced to previous studies , as follows: a) normal overall cognitive function just like aMCI; b) the tests scores about memory function are in the normal range while deficits were present on other cognitive domains including visual spatial function, executive function, language function, and information processing speed; and (c) subjects aged between 50 and 80 years old.

Inclusion criteria of aMCI subjects were identified meeting the diagnostic criteria defined by Peterson et al. as well as the revised consensus standards presented by Winblad et al. , and the detailed inclusion criteria have been described in our previously published studies , as follows: (a) memory complaint preferably corroborated by an informant or the subject for more than 3 months; (b) objective memory impairment adjusted for age and educational level; (c) normal general cognitive function of MMSE score equal or above 24; (d) no or minimal impairment in daily living activities; (e) CDR=0.5; (f) subjects aged between 50 and 80 years old; and (g) absence of dementia symptoms that were not sufficient to meet the criteria of the National Institute of Neurological and Communicative Disorders and Stroke or the AD and Related Disorders Association criteria for AD.

Inclusion criteria of CN subjects was identified meeting the following rules: (a) without memory complaint; (b) normal cognitive performance matched with age and education; (c) CDR=0; (d) MMSE ≥ 26; and (e) subjects aged between 50 and 80 years old. .

The detailed exclusion criteria for all subjects have been described in our previously published studies , as follows: (a) a past history of stroke (modified Hachinski Ischemic Scale Score of > 4), alcoholism, head injury, brain tumors, Parkinson’s disease, epilepsy, encephalitis, major depression (excluded by HAMD), or other neurological or psychiatric illness (excluded by clinical assessment and case history); (b) major medical illness (e.g., cancer, anemia, thyroid dysfunction, syphilis, or HIV); (c) severe visual or hearing loss; (d) unable to complete neuropsychological tests or with a contraindication for MRI, and (5) T2-weighted MRI showing major white matter (WM) changes, infarction, or other lesions (two experienced radiologists analyzed the scans). All patients had no any medications.

# References

**Cedres, N., Machado, A., Molina, Y., Diaz-Galvan, P., Hernandez-Cabrera, J. A., Barroso, J., Westman, E. & Ferreira, D.** (2019). Subjective Cognitive Decline Below and Above the Age of 60: A Multivariate Study on Neuroimaging, Cognitive, Clinical, and Demographic Measures. *J Alzheimers Dis* **68**, 295-309.

**Chen, J., Chen, G., Shu, H., Chen, G., Ward, B. D., Wang, Z., Liu, D., Antuono, P. G., Li, S. J., Zhang, Z. & Alzheimer's Disease Neuroimaging, I.** (2019a). Predicting progression from mild cognitive impairment to Alzheimer's disease on an individual subject basis by applying the CARE index across different independent cohorts. *Aging (Albany NY)* **11**, 2185-2201.

**Chen, J., Chen, R., Xue, C., Qi, W., Hu, G., Xu, W., Chen, S., Rao, J., Zhang, F. & Zhang, X.** (2022). Hippocampal-Subregion Mechanisms of Repetitive Transcranial Magnetic Stimulation Causally Associated with Amelioration of Episodic Memory in Amnestic Mild Cognitive Impairment. *J Alzheimers Dis* **85**, 1329-1342.

**Chen, J., Ma, N., Hu, G., Nousayhah, A., Xue, C., Qi, W., Xu, W., Chen, S., Rao, J., Liu, W., Zhang, F. & Zhang, X.** (2020). rTMS modulates precuneus-hippocampal subregion circuit in patients with subjective cognitive decline. *Aging (Albany NY)* **12**.

**Chen, J., Shu, H., Wang, Z., Zhan, Y., Liu, D., Liao, W., Xu, L., Liu, Y. & Zhang, Z.** (2016). Convergent and divergent intranetwork and internetwork connectivity patterns in patients with remitted late-life depression and amnestic mild cognitive impairment. *Cortex* **83**, 194-211.

**Chen, J., Shu, H., Wang, Z., Zhan, Y., Liu, D., Liu, Y. & Zhang, Z.** (2019b). Intrinsic connectivity identifies the sensory-motor network as a main cross-network between remitted late-life depression- and amnestic mild cognitive impairment-targeted networks. *Brain Imaging Behav*.

**Dunn, C. J., Duffy, S. L., Hickie, I. B., Lagopoulos, J., Lewis, S. J., Naismith, S. L. & Shine, J. M.** (2014). Deficits in episodic memory retrieval reveal impaired default mode network connectivity in amnestic mild cognitive impairment. *Neuroimage Clin* **4**, 473-80.

**Hao, L., Wang, X., Zhang, L., Xing, Y., Guo, Q., Hu, X., Mu, B., Chen, Y., Chen, G., Cao, J., Zhi, X., Liu, J., Li, X., Yang, L., Li, J., Du, W., Sun, Y., Wang, T., Liu, Z., Liu, Z., Zhao, X., Li, H., Yu, Y., Wang, X., Jia, J. & Han, Y.** (2017). Prevalence, Risk Factors, and Complaints Screening Tool Exploration of Subjective Cognitive Decline in a Large Cohort of the Chinese Population. *J Alzheimers Dis* **60**, 371-388.

**Jessen, F., Amariglio, R. E., van Boxtel, M., Breteler, M., Ceccaldi, M., Chetelat, G., Dubois, B., Dufouil, C., Ellis, K. A., van der Flier, W. M., Glodzik, L., van Harten, A. C., de Leon, M. J., McHugh, P., Mielke, M. M., Molinuevo, J. L., Mosconi, L., Osorio, R. S., Perrotin, A., Petersen, R. C., Rabin, L. A., Rami, L., Reisberg, B., Rentz, D. M., Sachdev, P. S., de la Sayette, V., Saykin, A. J., Scheltens, P., Shulman, M. B., Slavin, M. J., Sperling, R. A., Stewart, R., Uspenskaya, O., Vellas, B., Visser, P. J., Wagner, M. & Subjective Cognitive Decline Initiative Working, G.** (2014). A conceptual framework for research on subjective cognitive decline in preclinical Alzheimer's disease. *Alzheimers Dement* **10**, 844-52.

**Petersen, R. C., Smith, G. E., Waring, S. C., Ivnik, R. J., Tangalos, E. G. & Kokmen, E.** (1999). Mild cognitive impairment: clinical characterization and outcome. *Arch Neurol* **56**, 303-8.

**Winblad, B., Palmer, K., Kivipelto, M., Jelic, V., Fratiglioni, L., Wahlund, L. O., Nordberg, A., Backman, L., Albert, M., Almkvist, O., Arai, H., Basun, H., Blennow, K., de Leon, M., DeCarli, C., Erkinjuntti, T., Giacobini, E., Graff, C., Hardy, J., Jack, C., Jorm, A., Ritchie, K., van Duijn, C., Visser, P. & Petersen, R. C.** (2004). Mild cognitive impairment--beyond controversies, towards a consensus: report of the International Working Group on Mild Cognitive Impairment. *J Intern Med* **256**, 240-6.

**Xue, C., Yuan, B., Yue, Y., Xu, J., Wang, S., Wu, M., Ji, N., Zhou, X., Zhao, Y., Rao, J., Yang, W., Xiao, C. & Chen, J.** (2019). Distinct Disruptive Patterns of Default Mode Subnetwork Connectivity Across the Spectrum of Preclinical Alzheimer's Disease. *Front Aging Neurosci* **11**, 307.

**Yan, T., Wang, W., Yang, L., Chen, K., Chen, R. & Han, Y.** (2018). Rich club disturbances of the human connectome from subjective cognitive decline to Alzheimer's disease. *Theranostics* **8**, 3237-3255.
